# Ivermectin Treatment for *Strongyloides stercoralis–*Associated Recurrence of Nongastric MALT Lymphoma

**DOI:** 10.1155/crh/7226796

**Published:** 2026-06-20

**Authors:** Ani Misirian, Cameron M. Quon, Michael Maranzano, Eric Tam

**Affiliations:** ^1^ Internal Medicine, Los Angeles General Medical Center/University of Southern California, Los Angeles, California, USA; ^2^ Hematology, Los Angeles General Medical Center/University of Southern California, Los Angeles, California, USA

## Abstract

A healthy 64‐year‐old male originally from Peru presented with fever and abdominal pain and was successfully treated for *Campylobacter* colitis. An interval colonoscopy revealed mucosa‐associated lymphoid tissue (MALT) lymphoma initially in remission after four doses of rituximab and radiation with 24 Gy in 12 fractions. Two years after treatment, his symptoms returned, with a workup revealing recurrence of his MALT lymphoma. Biopsies revealed an underlying duodenal *Strongyloides* infection. In lieu of systemic chemotherapy, the patient was treated with oral ivermectin, which resulted in a complete response. MALT lymphoma is an indolent malignancy that can respond to treatment of an underlying infectious cause, and clinicians should be mindful of broad infectious etiologies, especially in immigrant populations.

## 1. Background

Mucosa‐associated lymphoid tissue (MALT) lymphoma is an indolent malignancy that is often driven by an underlying infectious process such as *H. pylori, Campylobacter,* and Chlamydia *psittaci (C. psittaci)* [1]. Many cases of gastric MALT lymphoma are attributable to *Helicobacter pylori* (*H. pylori*) infection and are responsive to eradication of infection. An association between nongastric MALT lymphoma and *Strongyloides* infection is unclear.

## 2. Case Report

The patient first presented as an otherwise healthy 64‐year‐old male originally from Peru with one week of fever, abdominal pain, body aches, and constipation with overflow diarrhea. He was admitted for sepsis with fever, tachycardia, leukocytosis, and a computed tomography (CT) scan showing diffuse thickening of the rectum and sigmoid colon with inflammatory stranding. His stool culture was positive for *Campylobacter* species. His symptoms resolved after a course of metronidazole, ciprofloxacin, and azithromycin, with a plan to perform an outpatient colonoscopy.

**FIGURE 1:**
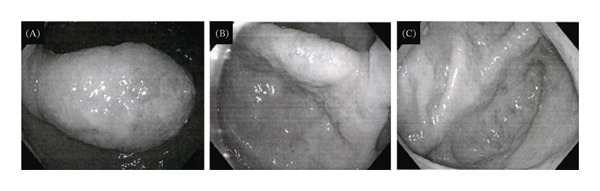
Initial colonoscopy showing (A) a mass in the ascending colon with (B) thickening of the colonic folds and (C) inflammation in the cecum.

**FIGURE 2:**
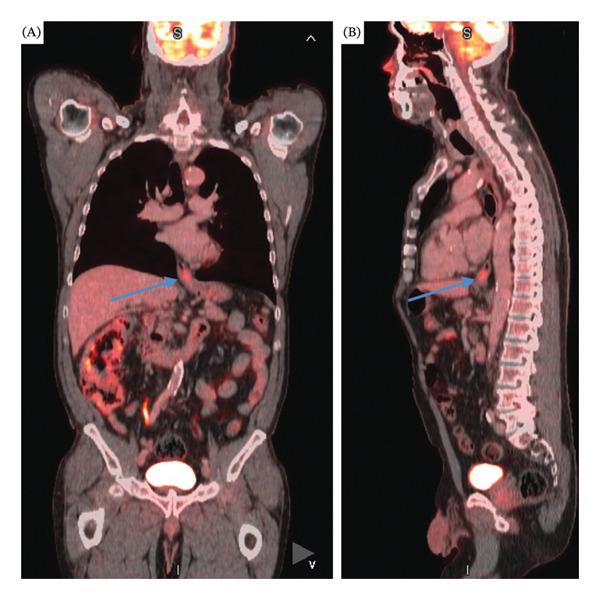
Initial positron emission tomography (PET) scan showing increased fluorodeoxyglucose‐^18^F (FDG) activity in the distal esophagus in the (A) axial and (B) sagittal planes as indicated by the blue arrow. This finding prompted an esophagogastroduodenoscopy (EGD).

The patient underwent a colonoscopy 6 months after his hospital admission. In the ascending colon, there was a 3 cm lesion encompassing an entire fold with overlying inflammation in addition to erythema and friability in the cecum (Figure 1). Pathology of these areas was consistent with extranodal marginal zone lymphoma of MALT with plasmacytic differentiation (MALT lymphoma) with B‐cell gene rearrangement that was IgK and IgH positive. Given these findings, he underwent a positron emission tomography (PET) scan, which showed fluorodeoxyglucose‐^18^F (FDG) activity in the distal esophagus (Figure 2). Therefore, he underwent an esophagogastroduodenoscopy (EGD) with biopsies showing chronic gastritis, intestinal metaplasia, and chronic nonspecific duodenitis.

At the time of his initial diagnosis, the patient was negative for *H. pylori* and HIV. However, he had B‐cell gene rearrangements in both the immunoglobulin kappa and immunoglobulin heavy chains. Two years after achieving a complete response to four doses of weekly rituximab infusions, our patient was found to have an asymptomatic recurrence of his lymphoma in the ascending colon and cecum on surveillance colonoscopy. He received radiation with 24 Gy in 12 fractions, with subsequent colonoscopies four months after radiation demonstrating no residual evidence of disease.

**FIGURE 3:**
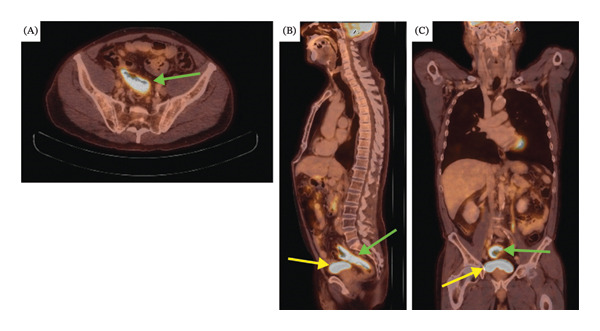
Repeat PET scan performed due to recurrence of symptoms. Images show increased FDG uptake in the colon as seen across the (A) coronal, (B) sagittal, and (C) axial planes. The green arrows point to the areas of colonic uptake. The yellow arrows indicate the normal activity of the bladder.

**FIGURE 4:**
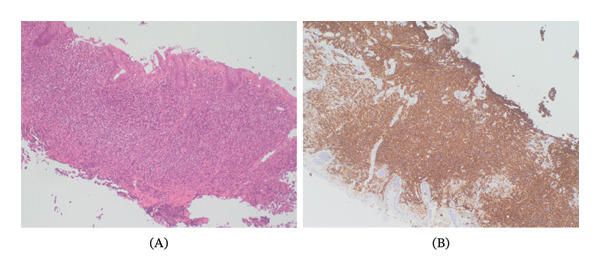
(A) Mature B‐cell lymphoma in the descending colon (H&E, 20×). (B) CD20 membranous staining in lymphoid cells.

**FIGURE 5:**
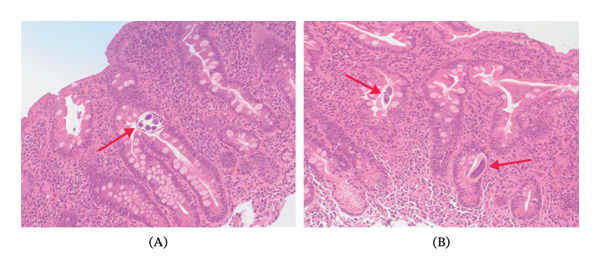
Strongyloides in small bowel mucosa (H&E, 40×). (A) and (B) Two different biopsies of the duodenum with helminths in duodenal crypts as indicated by the red arrows.

One year after achieving a complete response to radiation, the patient developed persistent periumbilical abdominal pain reminiscent of his initial symptoms. A repeat PET scan revealed new rectosigmoid circumferential wall thickening with hypermetabolic activity (see Figure 3), prompting another EGD and colonoscopy. While the EGD demonstrated normal mucosa throughout the esophagus, stomach, and duodenum, the colonoscopy showed nodular and friable mucosa throughout the transverse and descending colon, concerning for malignancy. Pathology revealed MALT lymphoma in the transverse colon, descending colon, and rectum (Figure 4). On the duodenal biopsy, there were helminth larvae in the duodenal crypts suggestive of *Strongyloides* species, with surrounding normal duodenal mucosa (Figure 5). Serologies were positive for *Strongyloides* IgG, though stool ova and parasite testing were negative.

Of note, the patient had mild eosinophilia (between 0.6K and 1.6K, range of normal between 0 and 0.4K) since the time of his initial diagnosis, which did not resolve in response to prior therapies. Systemic therapy for the patient’s lymphoma recurrence was deferred in favor of treating the patient’s *Strongyloides* infection with four total doses of oral ivermectin 200 mcg/kg daily, divided into two sets of daily dosing two weeks apart. This treatment strategy of four doses, instead of the guideline‐recommended one dose, was dosed to stop the autoinfection cycle in our patient who had previously received rituximab. The patient reported improvement of symptoms with the completion of ivermectin. A repeat PET scan completed 3 months after completion of ivermectin therapy revealed resolution of hypermetabolic activity, indicating complete response to ivermectin.

An interval endoscopy performed 2 months after the PET showed some atrophic gastric mucosa consistent with Barrett’s esophagus and mild duodenitis. Pathology showed no evidence of lymphoma or infectious organisms.

He continues to follow up at our medical center for disease surveillance.

## 3. Discussion

In cases that are caused by *H. pylori*, MALT lymphoma response rates can be as high as 75%–80% with treatment of infection, while cases caused by *C. psittaci* can have up to 65% lymphoma response rate after antibiotic therapy [2]. *H. pylori* is implicated in most gastric MALT lymphomas but does not have the same strong association with nongastric MALT lymphomas [2].

While rare, *Strongyloides* infection as a cause of MALT lymphoma has been reported in isolated cases in the existing literature. *Strongyloides* is a helminth that is endemic to many parts of the tropics and subtropics, including Asia, South and Central America, and Sub‐Saharan Africa. Worldwide, more than 2.6 billion people are estimated to be at risk of *Strongyloides* infection [4]. Transmission occurs via direct larval penetration of human skin through soil. In the United States, most cases of *Strongyloides* infection are found in immigrants and in those from endemic regions. Infections can be acute or chronic, and immunocompetent individuals often present with nonspecific gastrointestinal symptoms [5]. Chronic infections can be maintained for decades through autoinfective cycles, and in immunocompromised individuals, they can lead to lethal hyperinfection syndrome [6]. The treatment of choice for *Strongyloides* infection is a single dose of the anthelminthic ivermectin, which has better efficacy and less adverse events than albendazole [7]. In our patient, we elected to give a total of four doses of ivermectin to interrupt the autoinfection cycle, since our patient had previously received rituximab. Because of the rarity of *Strongyloides* as an etiology, it took 5 years to identify his infection. The refractory nature of his disease course prompted repeat imaging and an EGD that led to the diagnosis.

Synchronous cases of gastric and colonic MALT lymphoma have been reported in patients with simultaneous *H. pylori* and *Strongyloides* infection, which regressed after eradication of both pathogens [8, 9]. Case reports have also reported gastric adenocarcinoma found in a patient with *Strongyloides stercoralis* infection, as well as colorectal adenocarcinoma in a patient with chronic *S. stercoralis* infection, suggesting that *Strongyloides* infection could trigger a host immune response that predisposes to carcinogenesis [10].

Treatment options for MALT lymphoma depend on the primary organ and degree of dissemination. For patients with localized symptomatic extra‐gastric MALT lymphoma, radiation therapy is used or is also offered to those who do not respond to initial *H. pylori* or other infectious eradication therapies [2, 11]. Those with unresectable lymphoma or in cases where radiation is contraindicated can be offered single‐agent rituximab in Stage I or Stage II disease [2]. In Stage II disease or higher, first‐line therapies include 4‐weekly doses of single‐agent rituximab or chemoimmunotherapy with bendamustine and rituximab [12]. Second‐line options for MALT lymphoma include indefinite BTK inhibitors acalabrutinib or zanubrutinib, for which side effects include cytopenia and infection [13].

## 4. Conclusion

Extranodal MALT lymphoma is a rare entity with many infectious causes. Identification and treatment of infectious causes, such as *Strongyloides,* can lead to a complete response without the need for further systemic therapy. Less common endemic infections should be considered, especially when treating immigrant populations. Here, we described a case of recurrent extranodal MALT lymphoma in a patient with *Strongyloides* infection, who, having previously been treated with systemic immunotherapy and radiation, was able to avoid further systemic therapy with BTK inhibitors or chemotherapy. Further research is necessary to determine the causative link between *Strongyloides* infection and extranodal MALT lymphoma onset.

## Funding

No funding was received for this study.

## Consent

Informed consent was obtained by the authors.

## Conflicts of Interest

The authors declare no conflicts of interest.

## Data Availability

Data sharing is not applicable to this article as no datasets were generated or analyzed during the current study.
